# An EEG-Based Neuromarketing Approach for Analyzing the Preference of an Electric Car

**DOI:** 10.1155/2022/9002101

**Published:** 2022-03-18

**Authors:** Somayeh Raiesdana, Morteza Mousakhani

**Affiliations:** ^1^Faculty of Electrical, Biomedical and Mechatronics Engineering, Qazvin Branch, Islamic Azad University, Qazvin, Iran; ^2^Department of Public Administration, Science and Research Branch, Azad University, Tehran, Iran

## Abstract

This study evaluates consumer preference from the perspective of neuroscience when a choice is made among a number of cars, one of which is an electric car. Consumer neuroscience contributes to a systematic understanding of the underlying information processing and cognitions involved in choosing or preferring a product. This study aims to evaluate whether neural measures, which were implicitly extracted from brain activities, can be reliable or consistent with self-reported measures such as preference or liking. In an EEG-based experiment, the participants viewed images of automobiles and their specifications. Emotional and attentional stimuli and the participants' responses, in the form of decisions made, were meticulously distinguished and analyzed via signal processing techniques, statistical tests, and brain mapping tools. Long-range temporal correlations (LRTCs) were also calculated to investigate whether the preference of a product could affect the dynamic of neuronal fluctuations. Statistically significant spatiotemporal dynamical differences were then evaluated between those who select an electric car (which seemingly demands specific memory and long-term attention) and participants who choose other cars. The results showed increased PSD and central-parietal and central-frontal coherences at the alpha frequency band for those who selected the electric car. In addition, the findings showed the emergence of LRTCs or the ability of this group to integrate information over extended periods. Furthermore, the result of clustering subjects into two groups, using statistically significant discriminative EEG measures, was associated with the self-report data. The obtained results highlighted the promising role of intrinsically extracted measures on consumers' buying behavior.

## 1. Introduction

Electric vehicles are seen as a promising technology since they can help reduce carbon emissions and move toward more sustainable transport [1]. The popularity of electric vehicles has been considerably increasing around the world over the past few decades. However, in a highly traditional society like Iran, it is extremely challenging to adopt electric cars and replace classic cars with them. In fact, cultural, social, and economic factors heavily influence decisions for buying a suitable vehicle [2].

In the Iranian market, industry owners and manufacturers should consider social and psychological issues during the production process or even before that since improvements in technology by itself will not be sufficient for market success. Analyzing the consumers' behaviors and their brain functioning at liking/disliking or preferring/not preferring a product to purchase can be considered as a new horizon in marketing analysis. To examine consumers' minds or their preferences in neuromarketing, it is common to investigate a group of society members at the conscious or even unconscious level to understand how they behave or their brains function during the selection/purchase process. This analysis will help predict consumers' future choices and improve product manufacturing based on consumers' demands [3].

Neuromarketing is a newly emerging interdisciplinary field that analyzes consumers' behaviors from the viewpoint of the human brain. Neuromarketing is at the intersection of neuroscience, psychology, and marketing and focuses on consumers' emotional and cognitive responses to marketing stimuli. The main objective of this field is to effectively transfer commercial messages to consumers and increase the purchasing likelihood [4, 5]. Interestingly, this field provides an opportunity to directly extract hidden, reliable information from customers' brains, reflecting their emotions or cognitions without making any inquiries. Researchers believe that the subconscious mind has a more significant impact on human life and people's choices than the conscious mind. The hypothesis in neuromarketing is that the intellectual structure of consumers' activities mainly resides in the subconscious area of the brain with relatively little controlled consciousness. Therefore, technologists who study the role of perceptions in the market tend to learn valuable techniques to effectively manipulate the subconscious activities of the brain [6].

In an attempt to model customers' mental map in neuromarketing, six determinative marketing stimuli were introduced by [7]. Self-centered desire states that customers unconsciously think about the benefits that they acquire by selecting a specific product. The contrast between products and their specifications is an essential factor that helps to finalize the decision process. Tangible input helps an incredulous and analytic mind to make decisions quickly. The initial and final content of a stimulus is more remembered. Visual stimuli are effective for making fast and sure decisions. Emotion persuasion helps to reminiscent and cause reactions which are very influential in customer decision-making [7].

**Figure 1:**
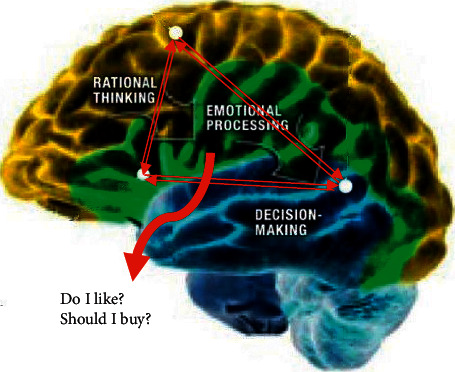
Active brain regions and connections in marketing neuroscience.

Brain activity in neuroscientific research can be measured by a variety of tools, including Positron Emission Tomography (PET), Evoked Response Potential (ERP), Magnetoencephalography (MEG), Functional Magnetic Resonance Imaging (fMRI), and EEG. Electrooculogram (EOG) signal recording, facial coding, and eye-tracking can further characterize cognitive activities. In neuromarketing, the activities of different brain regions involved in processes related to buying decisions are measured and analyzed [8]. When a consumer views a product, the higher activation of neural regions indicates the higher probability that the consumer will purchase the given product. Experiments on neural activities with fMRI and EEG indicated that three distinct neural regions, including neocortex (new brain), limbic system (middle brain), and brain stem (old brain), are responsible for making decisions in neuromarketing. These regions are demonstrated in Figure 1. The neocortex is associated with “higher” functions of the brain, including information processing of the sensory perception, conscious movements, reasoning, plan, decision-making, and language. The limbic system, often referred to as the emotional brain, is a group of structures in the brain involved with motivation, emotion, enjoyment, and memory. The old brain located in the brainstem and midbrain underneath the limbic system is the most critical region in making decisions [7].

A review of studies with significant findings in marketing neuroscience is presented in the following. In addition, a comparative review has been tabulated in Table 1 summarizing some significant findings which were utilized to scheme the analysis procedure in this paper. Several studies have attempted to increase the effectiveness of advertising messages to increase product sales by reviewing and analyzing consumers' brain activities [9].

EEG has been widely used for this analysis due to its simplicity, availability, low cost, and informativeness. EEG analyses have provided empirical evidence indicating that certain aspects of consumers' cognition and emotional responses to advertisement messages (even below the level of conscious awareness) can be successfully monitored and characterized in real time. In research [10], frontal EEG activity changes were traced while observing commercial video clips. Power spectral density maps showed an asymmetrical increase of theta and alpha activities related to observing pleasant/unpleasant advertisements in the left/right hemisphere. This work distinguished the “like” and “dislike” datasets by different EEG power spectral maps in theta and alpha bands [10]. In another study [11], an analysis was performed using both cognitive and behavioral measures to demonstrate the effectiveness of the advertisement as a result of a particular gesture by a female model. Participants were asked to watch a series of advertisements while their EEG, EMG, and skin conductance responses were registered, which enabled identifying different neurophysiological patterns of brain functioning and facial muscles connected with emotions and arousal. The results showed that different marketing stimuli caused differences in consumers' neurophysiological reactions even though the differences were not seen consciously; that is, people did not recognize any differences between the stimuli at the conscious level [11]. In a similar study [12], frontal cortex activation in reaction to TV advertisements was assessed. Three different Sony Bravia commercials, which were designed to show the uniqueness of color for this product, were tested by five competent advertising experts. The participants consistently identified two different parts of the advertisement in terms of content type. The first part aimed to build emotional engagement, while the second part focused on presenting the product information. The emotional and informational parts served as independent variables for the test. The results of repeated measure ANOVA and post hoc tests demonstrated that dominant reactions were presented only in response to one of the tested advertisements [12]. In another study [13], the participants were requested to choose their preferred crackers described by shape, flavor, and topping to assess the brain response to marketing stimuli. Mutual information was utilized to quantify the importance of different cracker features contributing to the product design. The authors used brain source separation and wavelet denoising to clean the data, analyze power spectral for feature extraction, characterize the dynamics of ongoing EEG, and analyze mutual information to identify the associations between subjects' preferences and EEGs. They found apparent phase synchronization between the left- and right-frontal and occipital regions, which indicated interhemispheric communications during the chosen task [13]. In addition, alpha mutual information was quantified, which highlighted the impacts of different flavors and toppings than shapes attribute for preferring the crackers [13].

Another group of researchers tried to model consumers' choices which were often realized in terms of “likes” and “dislikes.” The relationship between brain imaging and consumers' decisions was investigated in [14] while subjects were watching a virtual video of a supermarket to select one of the three brands of a given product. A strong correlation was found between brain activation in the right parietal cortex and the subjects' familiarity with a brand. In another study using EEG and eye-tracking analysis [15], the decision-making process was analyzed when a set of pictures of products were presented to the participants. Like and dislike responses for images displayed on a computer screen were evaluated using the main frequency band analysis by Principal Component Analysis (PCA) and Fast Fourier Transform (FFT). Results showed changes in the spectral activity of theta bands in frontal, parietal, and occipital areas. Furthermore, a wireless EEG neuromarketing system with multimedia stimuli was implemented in [16] to characterize the preference for an automobile brand. The participants in this study (*N* = 12) were stimulated by advertisement videos.

The spectral changes in the alpha band were found via extracting power spectral density, spectral energy (SE), and spectral centroid (SC). The extracted features were fed to two different nonlinear classifiers, k-Nearest Neighbor (k-NN) and Probabilistic Neural Network (PNN), to classify the participants' responses to the advertisement. The best result of this research (accuracy of 96.62%) was achieved for the PSD feature with the PNN classifier. In another study, a hidden Markov model (HMM) classifier was utilized to predict the users' preferences [17]. EEGs of 40 participants were recorded and denoised with a Golay filter while viewing pictures of the products. The features were extracted using Discrete Wavelet Transform (DWT). An HMM-based classification was applied to predict consumers' choices. Users' choices provided the ground truth for such a prediction model. This model was learned with EEGs recorded from *N* − 1 subjects and tested with an EEG record of the *N*th subject. Results of the HMM-based choice prediction model were better than other tested classifiers such as the neural network. Within-sample and population level preferences were also predicted based on subjects' questionnaire responses and EEG measures extracted during the commercial viewings. It was also found that the most predictive EEG measures were frontal powers in the alpha band, hemispheric asymmetry in the beta band, and intersubject correlation in delta and alpha bands [18].

Based on the literature review, emotion, attention, and memory are the most commonly adopted variables in neuromarketing for understanding consumers' brain functions. It is hypothesized that a network of different brain regions supports human brain functioning/decision-making [19]. Neuromarketing researches have revealed that certain brain areas are responsible for various cognitive and mental functions in buying decision-making. The decision-making process generally recruits the concerted activity of several areas. The executive power of decision-making relies on extensive neuronal circuits and dynamical/causal interactions of functionally segregated regions [20]. The primary nodes of this network are cortical and subcortical regions with variable activities. These activities reflect the brain function when consumers are stimulated by the appearance and characteristics of products or when they are requested to express their preferences. Some studies have shown that consumers' preferences could be linked to the frontal brain regions, specifically the medial prefrontal cortex, nucleus accumbens, and medial orbitofrontal cortex [20]. Some other studies have demonstrated that the preference is linked to the frontal brain regions, specifically, the medial prefrontal cortex, nucleus accumbens, and medial orbitofrontal cortex [21].

The present study focuses on consumers' decision-making process via EEG signal analysis from the neuroscientific viewpoint when they make a tradeoff between the appearance and attributes of electric and nonelectric cars. This study aims to understand consumers' buying behavior and to evaluate the behavioral and neural correlates when subjects choose their preferences. We are going to investigate and compare consumers' self-report responses by EEG-based extracted responses when they are presented to emotional and attentional stimuli from nonelectric and electric automobiles. We try to distinguish choices made either emotionally (by viewing images of cars) or attentionally (by viewing images with information) via signal processing techniques, statistical tests, and brain mapping tools. It is further assessed whether introducing product specifications, that is, communicating the differentiating characteristics of the electric car to consumers, can affect the consumer choices or not. The neuromarketing task in this study is designed in such a way that participants are conducted to rely on product attributes or quality indicators in order to guide the decision-making of subjects and lead them to choose the electric car.

Based on the given explanation on regional neural modulation due to marketing emotional and attentional stimuli that are reflected in power, coherence, and dynamics of the brain, we perform power spectral density and coherence analysis at different regions, frequency band, and task stages. Furthermore, we pursue analysis based on the critical brain hypothesis or self-organized criticality, according to which brain networks operate at or near a critical state in order to maintain a flexible repertoire of responses to (marketing) stimuli. Fractal theory and self-similarity concept is used to conceptualize the contrast of predictability and complexity of costumers' behaviors during the stimulation/response intervals. Additionally, to evaluate the possibility that salient patterns in EEG recordings can predict the self-reported responses, we implement a clustering algorithm in order to predict consumers' choices. By combining discriminative EEG parameters and using a simple yet powerful clustering tool (the K-mean algorithm), relationships between the content of inner conscious experience and the electrical brain activity are also investigated. The correlation of self-report data and EEG parameters is assessed to determine whether relying on intrinsically extracted parameters beyond the self-report is possible or not.

The rest of the paper is organized as follows. In Section 2, materials and methods, including an experimental procedure for data acquisition and a review of the analysis methods, are presented.  Section 3 describes the implementation procedure in detail. In Section 4, the results are reported and discussed. Finally, a conclusion section states some major findings.

## 2. Materials and Methods

### 2.1. Experimental Design and Data Recording

In this study, EEGs were recorded using a 21-channel electroencephalograph apparatus (Neurowerk EEG, SIGMA Germany) at the biomedical research lab at the QIAU University. International 10–20 electrode placement standard was used, and the montage with referencing to ears was preset for recording. Channel locations were Fp1, Fp2, F3, F4, Fz, C3, C4, Cz, P3, P4, Pz, O1, O2, F7, F8, T3, T4, T5, T6, A1, and A2. The sampling rate was 256 Hz, and all signals were bandpass filtered at the frequency range of 0.3–70 Hz. Forty-five healthy voluntaries from QIAU students and staff members participated in the study. All participants were right-handed and in ages between 20 and 43 years, including 28 males (*m* = 34.4; SD = 5.83) and 17 females (*m* = 31.7; SD = 4.39). Subjects had no personal history of neurological or psychiatric disorders, and they were free from medications. Written informed consent, which was approved by the local institutional ethics committee, was received from each subject before the examination.

For EEG data recording, the participants were asked to sit in a comfortable position in front of a monitor. The experimenter explained the procedures for each subject before the test. Furthermore, written guidelines were presented in the first slide to provide some further instructions to the participants. It should be noted that the participants were not aware of the aim of the experiment in the beginning and did not know that an interview would be performed after recording.

Participants were exposed to automobile pictures on a computer screen in two separate stages. A number of consecutive slides, including images of different cars (and their specifications), were displayed on the screen to stimulate subjects emotionally (attentionally) at the first (second) stage of the experiment. The color of all cars was set to be white to eliminate the impact of color on selection of cars and also to prevent the contamination of color effect on cognitive effort or positive emotion. Each stimulation phase was followed by a decision-making slide in which all cars were shown from an identical view to allow the participants to make a choice. In this stage, the participants were asked to choose their preferred car among the five presented cars, one of which was an electric car. The participants' choices were recorded by pressing one of the five characters dedicated to each option.

**Figure 2:**
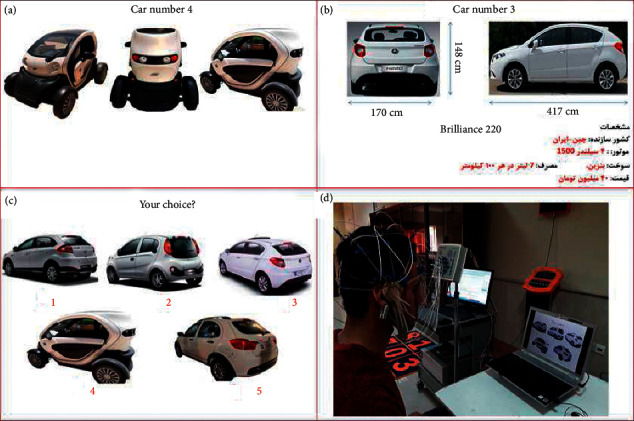
Demonstration of the experimental procedure. (a) A sample image shows the first stage of emotional stimulation. (b) A sample image shows the second stage of attentional stimulation (Persian written description is for Brilliance 220, made in China-Iran, 4-cylinder 1500 engine, fuel is petrol, fuel consumption is 7 liter per kilometer, and the price is 50 m toman). (c) Decision-making slide to wait for participant's response. (d) A picture of the experimental setup (the experiment was done at the Qazvin Islamic Azad University's (QIAU) research lab, May 2018, and the electric car, named YOZ, was designed by Syntec research team in this university).

In the emotion-based stage, each slide included three images of a car from different views (Figure 2(a)). Five different cars were displayed without any information. Images with no descriptions were displayed for 4 seconds. In the attention-based stage (i.e., the second stage), the procedure mentioned above was repeated so that each picture gave some information about the prominent characteristics of the corresponding car (Figure 2(b)). Images with descriptions in this stage were presented for 8 seconds. Additionally, waiting for making a decision at each stage lasted for 5 seconds (Figure 2(c)). After the test, each participant filled in a form and responded to some questions about his/her likes and preferences. This information was employed for behavioral analysis done in parallel with EEG processing.

### 2.2. Data Processing

**Figure 3:**
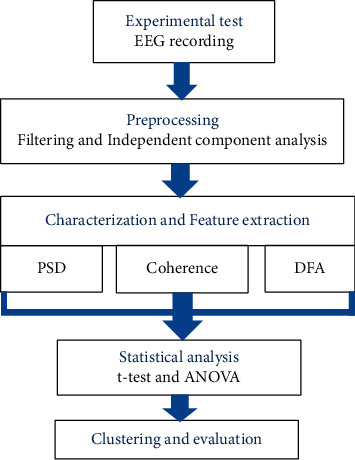
The block diagram of the methodology in this work.

Figure 3 shows the data processing steps in this study. Removing artifacts, characterizing signals, extracting features, and performing statistical evaluation tests are going to be applied to multichannel EEGs recorded while the subjects are stimulated within the designed neuromarketing platform. In the first step, the recorded signals are preprocessed with independent component analysis to denoise the signals and remove the existing artifacts. Artifacts, such as those generated by eye movements or muscular activities, as well as the artifacts outside the brain were removed using the ICA technique prior to any professional analysis of the data. Independent component analysis (ICA) is used to decompose the recorded scalp data into a set of cortical and artifact sources [28]. ICA can perform blind source separation for data matrix *X*, which is a mixture of sources. This algorithm finds an unmixing matrix (*W*) and yields the matrix (*U*) of independent component (IC) time courses when W is multiplied by the original data (*X*): *U*=*WX*.

This analysis satisfies the criteria that the time courses of resulting sources (rows in matrix *U*) are maximally independent. Inverting this equation by algebra yields *X*=*W*^−1^*U*. *W*^−1^ is a mixing matrix whose columns give relative weights for projecting the components to each scalp channel, that is, generating a scalp map for independent components. This method decomposes the cortical activity into its underlying sources, some of which are activity-related while others are noises or artifacts [29]. The sources detected as artifacts are removed by some analytical techniques, and the sources related to brain activity are preserved for further analysis.

The process of discriminating the sources is not straightforward since the activity of most sources, specifically those with unknown origins, are not stereotyped and stationary enough to be resolved as single sources. In addition, the signal strength of noisy and nonbrain components is small and represents mixtures of multiple sources. ICs related to artifacts can be detected by some rules of thumb. For example, eye movement sources are detected by their stereotyped scalp projections (they were reflected predominantly in frontal, frontocentral, and central electrodes in both hemispheres), or sources of muscle tension almost remain at the temporal locations. Furthermore, a systematic and operator-independent technique for source localization procedure can be employed after ICA decomposition. In this study, variance analysis is used to detect and preserve the dipoles with the least residual variance [30].

Then, a feature extraction step is performed to comprehensively characterize the data and its dynamic using a number of complementary methods. The changes in ongoing EEGs during emotional and attentional stimulations and decision-making phases are independently characterized in this study to be subjected to comparison. To this end, the frequency, phase, and dynamical contents of the purified EEGs as well as their subbands are quantified.

Power spectral density (PSD) measures the spectral power or the strength of variations of energy per unit of frequency which gives an estimate of signals' frequency content [31]. In this work, a nonparametric PSD estimation method (Welch's average modified periodogram) is used to compute the power spectra. In order to compute this measure, a time series is first divided into (possibly overlapping) segments, *a*(*n*). Then a window function, *w*(*n*), is applied to each of the segments. After applying the windowing functions, the modified periodogram of each segment is calculated as follows:

(1) $$\begin{array}{c} \hat{P}=\frac{1}{M}{\left\|\sum_{n=0}^{M-1}a_{i}\left(n\right)w\left(n\right)e^{-j2\pi fn}\right\|}^{2}. \end{array}$$

*M* is the number of segments. Finally, the Welch PSD estimate is calculated as the time average of the modified periodogram, which reduces the variance of the estimated PSD.

The next feature extraction method is coherence analysis. This analysis can demonstrate the similarity of the emergence of the oscillations, indicating functional corticocortical connections [31]. This method can analyze the underlying brain regions responsible for preference and information processing by computing coupling pattern. The aim of this analysis is to evaluate certain EEG frequency ranges to estimate the amount of temporal synchronicity and an in-phase pattern of oscillations in various spatially separated cortical areas [32].

EEG coherence represents the covariance of the spectral activity at two electrode locations [33]. EEG coherence is a measure for phase consistency as a function of frequency. In order to compute the coherence function *C*_*XY*_(*f*) at a frequency *f* for signals *X* and *Y*, the normalized cross spectrum is computed as

(2) $$\begin{array}{c} C_{XY}\left(f\right)=\frac{{\left|G_{XY}\left(f\right)\right|}^{2}}{G_{XX}\left(f\right)G_{YY}\left(f\right)}, \end{array}$$

where

(3) $$\begin{array}{c} G_{XY}\left(f\right)=\frac{1}{N}\sum_{n}F_{Xn}\left(f\right)F_{Yn}{\left(f\right)}^{*}. \end{array}$$

The numerator of (2) is the square root of the cross spectrum of the two EEG recording sites, and the denominator is the product of the power spectrum of the two individual sites. *F*_*Xn*_ is the Fourier transform of the signal *X* at epoch *n*, and *∗* indicates the complex conjugate series.

As the third signal characterizing tool, the emergence of long-range temporal correlations (LRTC) and power-law scaling behavior of the fluctuations in EEGs were quantified. Long-range temporal correlations in EEG oscillations are known as a signature of critical brain dynamics. Changes in LRTC are assumably associated with the underlying properties of neural activity and support the criticality hypothesis of the brain dynamics. Detrended fluctuation analysis (DFA) introduced by Peng et al. in 1991 [34] is used to detect intrinsic self-similarity/long-range correlations embedded in the nonstationary EEG time series. DFA can reveal scale-free behaviors and can reflect the tendency of complex systems to develop correlations, which decay more slowly and extend over more considerable distances in time and space [35]. Since the task in this study has different emotional, attentional, and decision-making stages and demands high memory and attention allocation, measuring the EEG persistence over time by dynamical measures (such as DFA) is proposed. DFA is a robust method for quantifying the long-range temporal correlations and estimating the power-law scaling exponent. The power-law scaling exponent describes the relationship between the averaged amplitude fluctuation (*F*(*t*)) and the temporal window size (the timescale) (*t*), that is, *F*(*t*) ~ *t*^*α*^. Computationally, *F*(*t*) was plotted for all window sizes on log-log axes. If a time series is characterized by long-range temporal correlations, *F*(*t*) increases with window size *t* according to the power law, and the DFA scaling exponent (or Hurst exponent) can be estimated from this log-log plot via linear regression as the slope *α*. Values of 0.5 < *α* ≤ 1 indicate the presence of persistent long-range temporal correlations, whereas *α* = 0.5 demonstrates randomly fluctuating oscillation amplitudes, which suggests that the data are completely uncorrelated. Furthermore, *α* = 1 (1/*f* noise) represents a compromise between the unpredictable randomness of white noise (*α* = 0.5) and the smoothness of Brownian noise (*α* = 1.5).

Next, a number of statistical tests are used to evaluate the behavioral and EEG-based measures. The significance of differences between groups and EEG measures is assessed and reported.

At the final step, the relationship between the content of inner conscious experience and brain electrical activity was evaluated. A k-means clustering technique was implemented to categorize the recorded EEGs into two clusters based on the statistically evaluated and verified features. A clustering method divides a dataset into groups according to similarities or dissimilarities among the patterns. Consumer responses are classified in this work using a KNN. We used the answers from the questionnaire as a baseline for the prediction attempt. K-means algorithm is one of the most straightforward and well-known unsupervised clustering algorithms, which aims to partition an observation into some cluster based on the mean of the observation. This algorithm determines the cluster centers and the samples belonging to them by minimizing a squared error-based objective function [36]. The k-means algorithm tries to locate the cluster centers far away from each other and associate each data point to the nearest cluster center. In general, the k-means algorithm searches for the k-partition calculating within-cluster sum of square by moving from one cluster to another [36].

## 3. Results

### 3.1. Results of Behavioral Analysis

**Figure 4:**
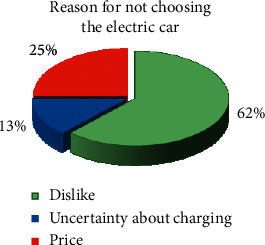
Pie plot of reasons for not choosing the electric car.

Table 2 shows the choices made by participants in the test of this study. The participants were assigned to groups A and B based on choosing/not choosing the electric car (the buttons pressed by subjects were analyzed to define which car was selected). In each group, they were further subdivided into two groups of emotion-based and attention-based according to self-report. In other words, subjects were divided into these groups based on the answers to questionnaire items asking their reasons for preferring/not preferring the electric car. Further, the number of subjects who changed their choices in the second stage of the test was determined.  Figure 4 shows the pie plot of reasons for not choosing the electric car. The main reason for not choosing the electric car was aesthetic factor (62% of those who did not select this car stated that they did not like it).

A few tests were performed to report the statistical significance of the consumers' preferences. First, it was verified that a randomly selected subset of samples from a regular population had a normal distribution. The normality of data distribution was checked and verified with Shapiro–Wilk (S-W) test. Then, a number of distinct inferential *t*-tests were run for “age,” “gender,” “car,” and “selection” variables. The level of statistical significance is expressed as a *p* value. A *p* value less than 0.05 (*p* < 0.05) is statistically significant. This indicates that the null hypothesis is rejected, and the alternative hypothesis is accepted.

The hypothesis for the first test was whether selecting products depends on age. The test showed significant age differences between the two groups (*p* < 0.01). Nonetheless, as evidence suggests that the brain rhythms and networks would change across the lifespan [37], it is possible that, in addition to subjects' preferences, their age contributed to the group differences in EEG measures. In order to elucidate these effects, an analysis of covariance test on EEG measures with the effect of age was accomplished, which is going to be reported in the next section when EEG measures are statistically evaluated. The second statistical test was conducted to evaluate the statistical significance of the hypothesis that whether selecting products depends on gender. The null hypothesis stating that “there are no statistically significant differences between men's and women's choices” was rejected with a 95% confidence interval at both stages of the study (*p* = 0.36 for emotion-based and *p* = 0.12 for attention-based).

Next, the participants' choices (electric or nonelectric cars) were statistically evaluated. In this test, the statistical significance of differences between groups A and B in the emotion-based stimulation stage as well as the attention-based stimulation stage was estimated using *t*-tests. The test hypothesis (i.e., the existence of significant differences among the number of selections of the electric car to all choices) was rejected when the subjects were stimulated only by photos of cars (*p* = 0.36). However, it was accepted when the subjects were stimulated attentionally (*p* = 0.04). This test confirms that, in the second stage of the task, selection of electric car is statistically different than the other types.

The final test in this section evaluates the significance of attentional selections in each group. We used the Chi-square test to assess if there exists an association between the choice of an electric or a nonelectric car and the usefulness of the given information at the second stage. This test was conducted concerning the question in the questionnaire: “do the informative descriptions at the second task affect your selection (Is the given information is useful enough to affect the choice results)?”

The hypothesis is that the proportion of the population with electric car selection, who answered “yes” to the question mentioned above, and the proportion of the population with nonelectric car selection and the “no” answer to that question are not different. The result of the Chi-square test showed that *χ*2 statistic = 13.47 (degree of freedom = 1) and *p* = 0.007. Thus, hypothesis H0 is rejected, indicating that knowledge of cars' specifications has a significant influence on the choices made by participants. This result states that the hypothesis of changing the preference after being informed by useful specification is verified.

### 3.2. Results of EEG Analysis

The processing steps mentioned in the previous section were all implemented with MATLAB software (version 2018b) on a Core i7b PC with 2G RAM. Prior to the primary processing, noises such as 50 Hz noise, unwanted interferences, and nonsignal artifacts were eliminated using a Butterworth 4th order bandpass filter with a cut-off frequency of 0.5–45 Hz.

**Figure 5:**
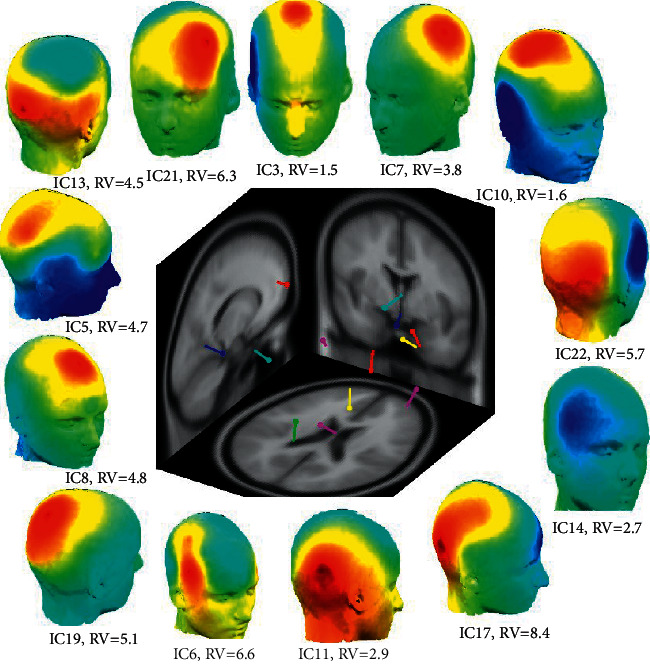
3D view of ICA decomposition for extracting EEG sources of recorded 21-channel data using EEGLAB. Dipoles are shown in different colors. The brain map for each component with an RV of less than 10% is also depicted.

ICA was implemented using the Runica algorithm in EEGLAB software (http://www.sccn.ucsd.edu/eeglab/), which is based on the Infomax algorithm. The ICA decomposition returned 21 independent components (ICs) for the recorded 21-channel EEG. Then, a scalp distribution or map was plotted for each subject (Figure 5). The extracted ICs were categorized into one of the following groups: cortical brain sources, physiological artifacts including eye movements and eye blinks, external artifacts including line noise, and spatially irregular components of unknown origin. In order to purify data, the least residual variance technique was implemented. The residual variance (RV) between the IC scalp map (W-1) and the model projection of the best-fit equivalent dipole was computed in EECLAB by estimating the error inherent to fitting sources within a spherical head model [38]. Inner brain sources (with variances less than 10%) are kept, while sources with higher variance were regarded as sources outside the brain and discarded. This procedure was performed for all subjects to exclude nonbrain sources and prune each subject's data. In Figure 5, the ICA decomposition is displayed in a 3D graphical view. Furthermore, the 3D scalp map for the remaining ICs and the corresponding residual variances written under each plot are demonstrated in this figure. After computing the RVs and deleting components with variances above the threshold, the purified EEG was recovered with ICA reconstruction using the reduced number of ICs.

After artifact rejection, the EEG record of each subject was subdivided into a number of segments using the timing of the stimuli and the intervals in between. Segments of emotional and attentional stimuli as well as the time intervals in between (given for making decisions) lasted for 20, 40, and 5 seconds, respectively. The data segments related to the emotional and attentional stimuli were further subdivided into five trials, each of which lasted for 4 seconds (for observing an image of a car) and 8 seconds (for observing an image of a car and its specifications).

**Figure 6:**
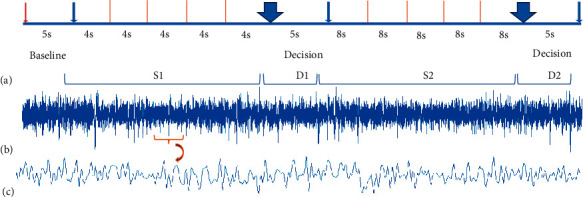
(a) The experiment's stages in sequence, including S1, D1, S2, and D2 segments. (b) A sample channel (c4) recording. (c) A few-second segment of the shown signal.

These segments were extracted from each record and entered into the analytical and statistical models. It is noteworthy that a five-second interval at the test initiation (before the stimulus start) was recorded as a baseline and was not directly considered for processing.  Figure 6 demonstrates the signal segmentation procedure.

#### 3.2.1. Results of the PSD Analysis

The cortical power spectral density was estimated for all frequency bands (delta = 1–4 Hz, theta = 4–8 Hz, alpha = 8–12 Hz, and beta = 12–30 Hz) as well as the whole frequency band that allowed us to investigate the frequency-dependent changes in the cortical SPD. A three-way ANOVA test, followed by post hoc Bonferroni test, was performed with frequency bands (delta, theta, alpha, beta, and whole), choice result (groups A and B), and task stages (S1, D1, S2, and D2) as factors to evaluate any interaction effects in PSD. The statistical threshold for significance was set at *p* < 0.05. Though the mean PSD for members of groups A, who selected the electric car, was higher compared to group B members (specifically at stages S1 and S2), quantitative analyses of PSD did not reveal a significant main effect of choosing the electric car (*F* (2; 582) = 86.40; *p* > 0.05). Furthermore, the PSDs computed for the alpha frequency band (and then for the theta frequency band) are higher among all investigated frequency bands during almost all stages for both groups. The results of the statistical analysis for PSD values of all frequency bands (delta, theta, alpha, beta, and whole) showed significant PSD for alpha frequency band (*F* (5; 498) = 92.8; *p* < 0.001). The other result of this test was the significance of frequency band × task stage interaction for attentional stimulation (S2) at *p* < 0.05 (*F* (9,699) = 103.7; *p* < 0.05). The increased PSD for attentional stimulation occurs at all stages, but it was significant for the alpha and theta band. The significance of PSD differences for attentional stimulation at alpha and theta bands outstands this stage of task where attention generates more power in brain rhythms.

For further analysis, the PSD values were computed in two frequency subbands (alpha and theta) as well as the whole frequency band for all segments of S1, D1, S2, and D2. The scalp maps based on 19 derivations are used to depict the results. In order to plot the maps, the values were color-coded and plotted at their corresponding positions on the planar projection. The values between the electrodes were interpolated using biharmonic spline interpolation. The details related to computing the PSD measure are as follows. The Welch method with Hamming window of 1 s, with an overlap of 0.5 s, was used to estimate the power spectral density of each signal segment. This choice represents a good compromise between the necessity to reduce the variance of the estimated spectrum and the need to reduce its bias. The number of FFT points was set to 256 to have a PSD estimate with a frequency resolution of 1 Hz (the frequency sampling is 256 Hz). The resulting PSD for each electrode was a feature vector of NF elements characterizing the power of the EEG oscillations from fL up to fH (the low and high-frequency values in each band). Notably, the whole frequency band was restricted to 1–40 Hz covering the entire spectrum reported in neuromarketing analyses.

**Figure 7:**
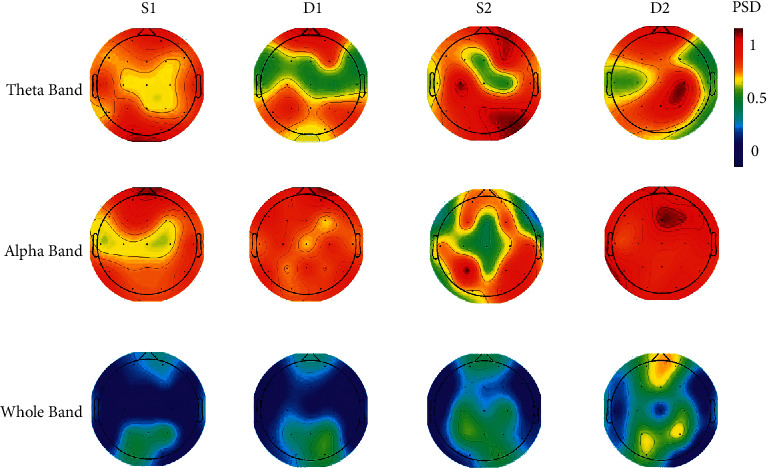
The scalp maps of PSD values for different frequency bands and different stages. Colors marked the normalized degree value of each corresponding electrode (blue denotes a low value and red denotes a high value).

Figure 7 shows the scalp maps representing the estimates of the PSD obtained by evaluating all channels in three different frequency bands and four different segments. Group-level results were obtained by cross-subject averaging. The maps in Figure 7 show less significant whole band activity. For the alpha band, the emotion-based stimulus has made higher activity in the frontal regions, while the attention-based stimulus is consistent with the parietal activity. On the other hand, the activity asymmetry in the theta band is more pronounced, and the attention-based stimulus has more theta activity. Additionally, the selection intervals D1 and D2 have activated frontal and occipital regions. These findings are in line with some previous studies examining the relationship between contextual preference and the medial orbitofrontal cortex. The higher activation in this region could be related to higher levels of preferences [39].

**Figure 8:**
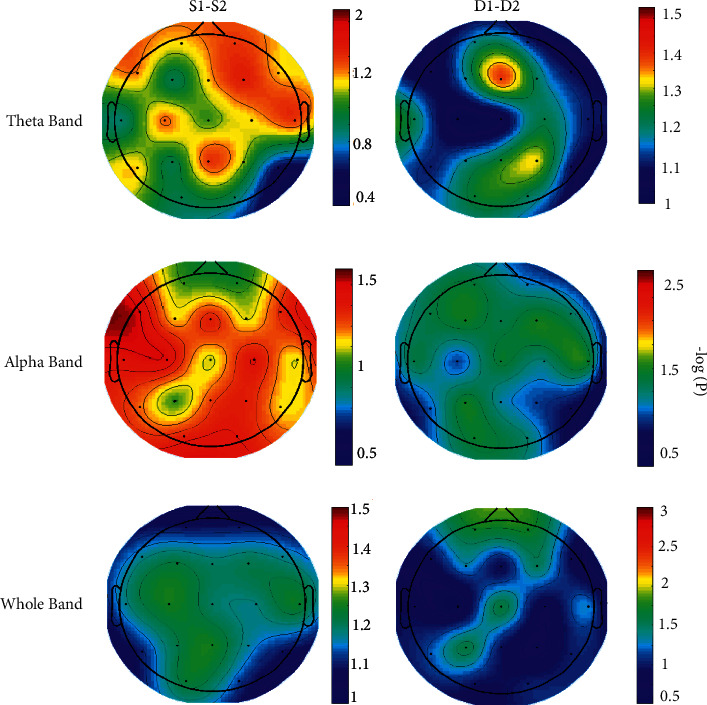
Topographical head maps representing *t*-values for *S*2 > *S*1 (first column) and for *D*2 > *D*1 (second column) for the theta band (first row), the alpha band (second row), and the whole frequency band (third row).

Statistical maps reporting topographic power differences between the two stimuli as well as the two decision conditions were further computed by paired *t*-tests and then plotted. Statistical comparisons were made between attentional-based stimulation and emotional-based stimulation (*S*2 > *S*1), as well as decisions made after attention-based stimulation and decisions made after emotion-based stimulation (*D*2 > *D*1). Log-transformed values were expressed in terms of *p* values over maps. In other words, the brain maps in Figure 8 were obtained by the logarithm of *p* values computed via contrasting the two conditions of the stimulus and the two conditions of the response.

The analyses were performed separately for the three frequency bands (corresponding to each row in Figure 7) on each electrode location. The resulting spectral maps highlighted the cortical areas in which the estimated PSD statistically differed between the two conditions at the level of *p* < 0.05; that is, the logarithm of *p* value greater than 1.3 (log(*p*)>−log(0.05)) in a channel indicated the statistical significance of stimulus/decision in that cortical position. As can be seen in the first column of Figure 8, the statistical maps show the predominance of theta activity in the right-frontal regions (Fp2, *t* = 2.1 and *p* = 0.05; F4, *t* = 3.47 and *p* = 0.044) and alpha activity in the central regions (Cz, *t* = 1.8 and *p* = 0.01; C4, *t* = 2.6 and *p* = 0.032) when stimulus S2 was compared to S1. No predominant activity was observed when the whole frequency band was analyzed.

However, when decision conditions were compared, less significant power differences were observed (right column). Furthermore, maps on the third row in Figure 8 show a lower (nonsignificant) power difference in centrooccipital regions, either in the stimulation (O1, *t* = 5.52 and *p* = 0.1; O2, *t* = –2.82 and *p* = 0.07) or in the decision (P3; Cz) but a relatively higher temporal activity in D2 in comparison to D1. Nonetheless, the comparison of D1 and D2 was not statistically significant (*t* = 6.5; *p* = 0.09). Overall, evaluating the maps in Figure 8 indicates that the alpha band can better contrast the attention-based stimulation and decision-making than emotion-based stimulation and decision-making.

#### 3.2.2. Results of the Coherence Analysis

Next, the coherence feature was computed to measure functional coupling among the cortical areas. Based on the results obtained in the previous test, the alpha frequency band was selected as the most discriminative frequency band. Electrodes of Fp1, Fp2, F3, Fz, F4, C3, Cz, C4, P3, Pz, P4, O1, and O2 were utilized for coherence analysis. Selecting electrodes was based on their higher power activity and the hypothesis that frontal, parietal, and occipital areas were engaged with emotion, attention, and decision-making cognition.

**Figure 9:**
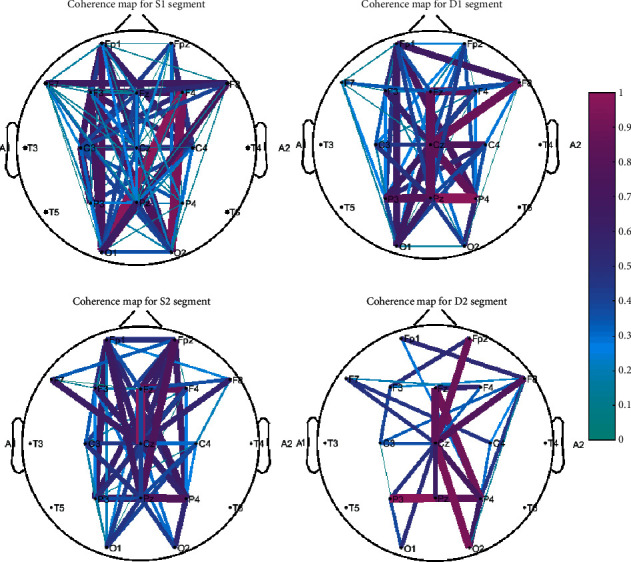
Topographical plots of coherence for S1, D1, S2, and D2 segments in the described task. The color of lines indicates the value of interelectrode coherence (a number in between 0 and 1).

To calculate the coherence matrix in this test, the EEGs were segmented into 10 s epochs using a moving window with 3 s overlap. The coherence measures were computed at electrode positions shown by bold dots in Figure 9. The coherence matrixes were visualized on the topographic coherence map by plotting the connections between the EEG electrodes, which vary in color and thickness to represent coherence coefficients. Maps were plotted for four conditions, including S1, S2, D1, and D2 (Figure 9). The results indicated coherent oscillation between frontal and central/parietal at segment S2 while subjects focused on the products' specifications. On the other hand, obvious coherent oscillations occurred between electrodes of Fz and Fp2 and parietal areas for the D2 segment, which indicated coupling between frontal and parietal regions when the subjects attempted to retrieve information from their memory. On the other hand, the linkage between occipital (O2; O1) and frontal (F3; F4) cortices for emotion-based neural activity can reflect a coherent association of vision and preference.

For further analysis, the intergroup brain activities were compared and analyzed in order to trace the differences in the coupling pattern.

**Figure 10:**
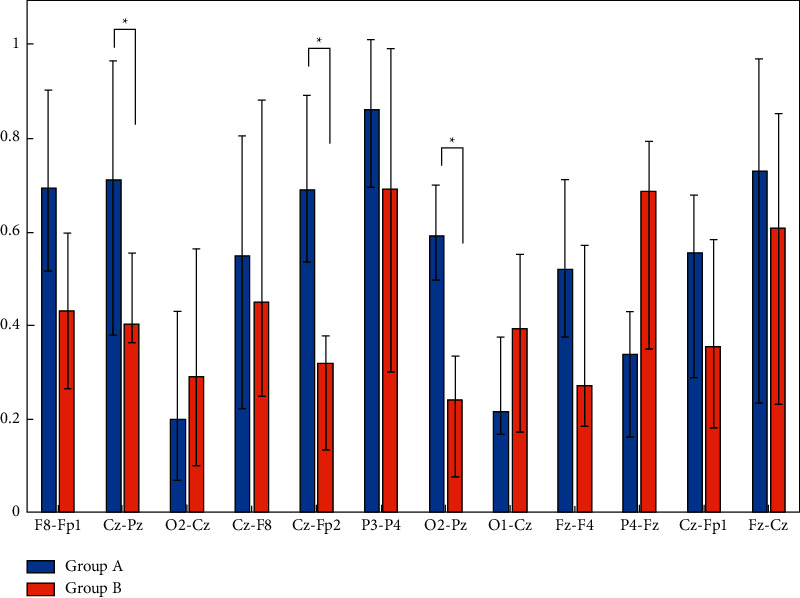
Statistical test (ANOVA) on coherence value for each pair of electrodes.

Maximum pairwise coherence values for all subjects in each group were computed, and an ANOVA statistical test was employed to find any statistically significant differences between the means of the groups. The test results showed a significant intragroup difference in terms of coherence (*p* < 0.05); that is, the EEG coherence difference between group A and group B in this work is significant. Another test was performed to specifically assess the statistical differences between interelectrode coherences. The average coherence values for all subjects in each group (16 subjects in group A and 29 subjects in group B) were computed on all pairs of the selected electrodes (Figure 10). Error bars with standard deviations are shown in Figure 10. Asterisks on the columns represent significant differences between the two groups with *p* < 0.05 level of significance in the ANOVA test. It is shown that central-parietal (Cz; Pz) and central-frontal (Cz-Fp2) coherences are statistically different among groups A and B; that is, those who selected the electric car have higher degrees of association or coupling of frequency spectra between central and parietal and frontal regions in their brains (A) in comparison to those who did not prefer the electric car (B). Overall, the higher coherence of group A members implies that they had highly activated visual attention, judgment, and information processing.

#### 3.2.3. Results of the DFA Analysis

**Figure 11:**
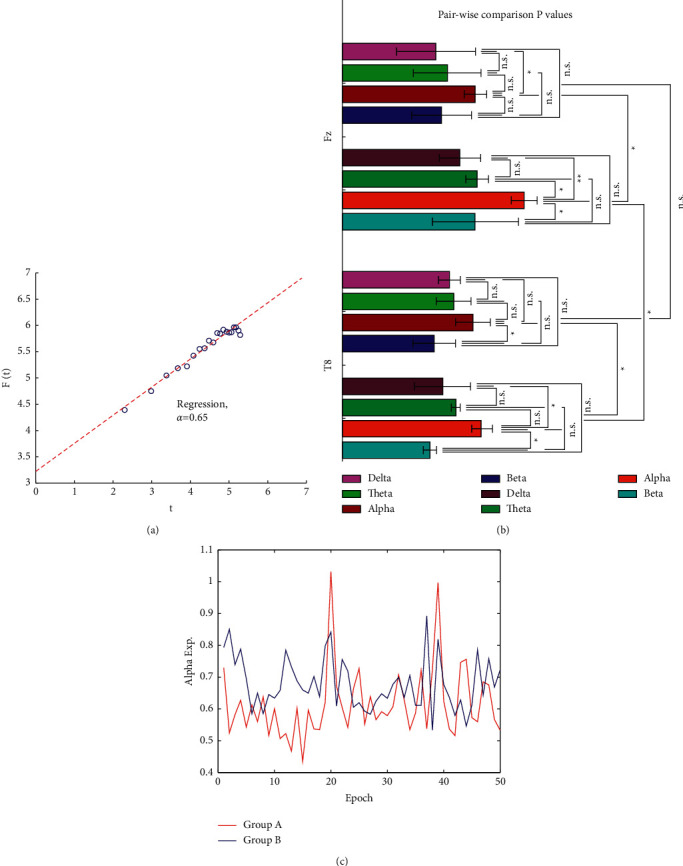
(a) Extraction of DFA exponent for a sample 4 s signal selected from S1 for a subject. (b) Mean and standard deviation of DFA values for selected channels in different frequency bands. At this test, the channels and the frequency bands which meet the higher level of statistical significance (*p* < 0.005) were selected. (c) Intergroup averaged-*α* computed for all consecutive 4 s signal segment for the C4 channel in each recorded data.

To calculate *α*, the amplitude envelopes of each signal were first extracted using the FIR bandpass filter and the Hilbert transform. Next, the root-mean-square fluctuation of the integrated and linearly detrended signals (*F* (*t*)) was calculated as a function of time window size (with an overlap of 50% between the windows) and plotted in double-logarithmic coordinates. The slope of the fluctuation function (*F* (*t*)) was estimated in a given interval, which was set as 2–20.  Figure 11(a) schematically shows the procedure of extracting the DFA exponent for a sample signal segment.

The statistical significance of differences between *α* exponent for the original time series and surrogate data was evaluated to assure that the dynamics of the recorded signal is deterministic. The purpose of surrogate data is to test for any nonlinearity in the original data. Surrogate data were generated to match the original signal. They had Fourier decomposition with the same amplitudes as the empirical data decomposition but with random phase components. In this test, ten surrogate data series were generated for each signal. Then the computed values of *α* for both series were subjected to a Wilcoxon signed-rank *t*-test to determine the significance of the test for the existence of nonlinearity. We found that the mean *α* for surrogate data and original data is distinct at the significance level of *p* < 0.05. Hence, the null hypothesis is rejected, indicating that the original data contains nonlinear features.

In order to investigate the effect of electrode position as well as the effect of frequency band selection on the measure of system dynamics or the brain scaling behavior, the average and variance of scaling exponents for all subjects in each group were computed at each frequency band and each electrode position and plotted as error bars. Pairwise comparison *t*-tests were further performed. As it can be seen for two sample channels in Figure 11(b), the average scaling exponents were significant in the alpha frequency band between groups. Figure 11(b) shows all pairwise statistical comparison between groups and frequency band. Comparison of alpha-beta and alpha-delta DFA was almost significant in both groups. However, differences of alpha-theta scaling exponents in all channels and in both groups were rarely significant. Furthermore, the results showed that the DFA measure is higher for frontal and then central recordings. The significance of results for the frontal region (Fz and Fp1) in comparison to temporal and parietal (*p* < 0.05) was observed for group B. That is, the long-range correlations estimated from frontal regions are extended on more scales. No more significant result is reportable for this text. Based on the results of this test, electrodes and frequency bands with a higher significance level (*P* < 0.005) were determined for later analysis. Frontal channels and the alpha frequency band better reflected the actual dynamics of the system and the groups' differences.

The overall variation of averaged *α* over all subjects in each group was also traced. The *α* fluctuation curves for groups A and B are computed for channel F8 in the alpha frequency band and illustrated in one plot (Figure 11(c)). As can be seen in this figure, LRTCs for both groups increased during decision-making periods. Additionally, long-range correlations for group A during attention-based stimulation and the subsequent decision-making period increased since most of the subjects in this group declared that they had selected the electric car when they read the specifications and became aware of the cars' capabilities.

At the end of this section, we assessed the effect of age on brain activity in the accomplished task. An exploratory analysis of the age effect was carried out by evaluating significant discriminative features detected at different stages, locations, and frequency bands. Two age groups were defined on the basis of participants' age range (younger below 25; older above 25). A repeated measure analysis of covariance (ANCOVA) was carried out to analyze the EEG changes for the younger and older between groups A and B. The repeated measures ANCOVA results confirm whether the sphericity and equal variance assumptions are satisfied. In this test, age was included as a covariate. In addition to explaining the effect size between the independent variable and the dependent variable, the interaction between group and age was analyzed and expressed as partial eta square (*η*2) at this test.

ANCOVA test was performed for each variable listed in Table 3. Each row in this table indicates a situation for which the results of signal processing were noticeable. Results showed a significant interaction between group and age when comparison was made with frontal alpha mean power of EEGs at stimulation stage (*F* = 3.18; *p* = 0.04). This interaction states that choices of subjects depend on age based on the changes that occurred in the power spectral density of their EEGs at the mentioned frequency band, channel location, and task stage. We had evaluated the age effect on subjects' selection at behavioral analysis in the previous section, which showed that there are significant age differences between the two groups. This test based on EEG measures showed that older subjects have chosen nonelectric cars significantly different than youngers. Other EEG analysis efforts based on coherence or DFA did not show any interaction between age (being younger or older) and the selection of car.

Nonetheless, it was observed that the effect size was small for frontal alpha mean power at stimulation sages and frontal alpha DFA at the decision-making stage, indicating a trend-level group by age interaction. Post hoc analysis showed that the group means differed significantly for frontal alpha DFA at the decision-making stage (*p* = 0.02). No significance in the main effect of age was observed.

### 3.3. Results of the Clustering Task

PSD, coherence, and scaling exponent values for all 10 s signal segments were computed in the given frequency band and channel location listed in rows of Table 4. These values were normalized and concatenated into one feature vector and then fed to a k-means clustering algorithm. The EEG data were labeled with self-reported data of all the participants to class A (electric car preference) and class B (nonelectric car preference). The number of clusters was *k* = 2, and two centroids were defined, one for each cluster. No seeds for the centroids were used. Therefore, two random feature points were chosen as the initial means. Two distance measures were used, including the squared Euclidean and the City Block for KNN implementation.

**Figure 12:**
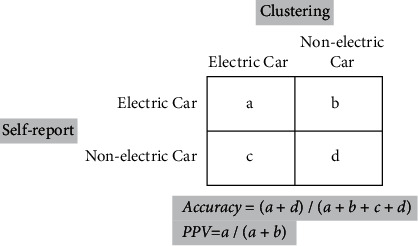
Clustering task to discriminate between groups A and B based on self-report.

The clustering procedure was implemented in two steps. Initially, cluster centroid was chosen randomly, and the distance of each data point from the centroid was calculated using the aforementioned distance measures. Then the new cluster centroid was calculated and these two steps iterated until no new centroids were produced. A 2 *∗* 2 confusion matrix associated with the classifier, which provided information about actual and predicted classifications, was constructed (Figure 12). Results of clustering for squared Euclidean distance measure were summarized in Table 2. Accuracy and Positive Predictive Value (PPV) for each clustering task were computed and tabulated. Furthermore, two statistical ANOVA tests were performed to analyze the clustering results. The hypothesis for the first test was H0: the centroids of the groups, as defined in the space of the chosen resemblance measure, are identical for both groups. The third column in Table 4 shows the *p* values for each test. Values less than the significant level at 0.05 indicate that there is a significant difference between groups. The centroids for groups with electric and nonelectric car selections were significantly not identical for the case when EEG measures were extracted from the alpha frequency band of central and frontal positions in the stages of D1 and D2. For some other rows of this table, the null hypothesis is rejected as well (rows 2, 4, and 5). The subsequent statistical test evaluated the hypothesis with H0 defined as follows: there is no difference between groups' dispersion. The analysis of multivariate homogeneity of group dispersions (variances) was performed with a beta diversity test. That is, the differences in the mean beta diversity between groups were assessed by ANOVA.

Results listed in the last column of Table 4 indicate *p* values for this test. *p* values less than 0.05 (rows 2 and 4 in Table 4) indicate significant differences between clusters. Finally, cluster analysis indicated that cluster 1 (C1) consisted of high PSD and coherence and less scaling exponent computed at alpha frequency band localized in frontal and central areas and more pronounced at simulation stages (D1 and D2). Cluster 2 (C2), on the other hand, was characterized by low PSD with relatively high *α* values.

## 4. Discussion

The differences between groups with electric/nonelectric car selection were characterized meticulously throughout the paper. Comprehensive characterization of these differences besides statistical evaluation was addressed to illustrate the changes that occurred in brain activity based on consumers' preference choice. Results of comparing the first and second stages of the test showed that subjects had changed their choice to the electric car when exposed to product attributes or product knowledge. Introducing intrinsic product attributes (fuel consumption, charge capacity, etc.) lets us communicate the superior quality of differentiating characteristics of the electric car to the consumer. Noteworthy, we conducted participants to rely on product attributes or quality indicators. This made it possible to guide the decision-making of subjects and lead them to choose the electric car that might not be preferred a priori by emotional stimulation. The reason for the less choice of the electric car in the first stage of this work mainly resided in aesthetic factors (see Figure 4 representing the questionnaire information). The potential to shift preference can provide advice for realigning marketing strategies.

A significant finding of this work is the observed differences between EEG measures of attention-based and emotion-based stimuli as well as the differences between the activated brain regions and the coherence patterns during the decision-making task for selecting a preferred product.

Maps related to the statistical test of power differences showed significant differences in brain regions responsible for emotion and judgment when the stimulus was emotional, as well as information processing and visual perception regions when the stimulus was attentional. Furthermore, EEG coherence measures represent the functional association between different brain regions, and they can evaluate the neurofunctional substrates underlying learning, reasoning, judgment, and decision-making. The altered connectivity patterns among brain sites for decisions made after emotional and attentional stimulations further confirmed that letting the subjects know about the specification and the advantages of an electric car, which may initially sound unfamiliar or strange to them, will change their brains activity and possibly their choices.

The dynamical analysis in this work showed memory-related changes in cerebral activity during the marketing task. Memory and attention processes were tracked by changes in long-range temporal correlations measured in EEGs as memory formation and memory retention were activated by visual stimulus over a range of time scales. Memory-related processes, such as brand awareness, product experience, and advertising recall, could influence consumers' behaviors. However, the subjects in this study had no long-term memory of the newly designed electric car. Their memory was just restricted to a medium/short-term memory of the introduced car and its specifications. Hence, the scaling behavior over longer time windows, which was observed for decision-making periods (D1 and D2), specifically when decisions were made after attention-based stimulation (D2), indicated that subjects tried to retrieve memories and use the information for judging and selecting their preferred products. It is noteworthy that a cognition-based selection process for an attention-demanding product is reasonably connected to the memorization process since the given information was successfully remembered in the subsequent interview when the participants claimed that they had chosen the electric car by paying attention to its specifications.

Using a global dynamic measure of the EEG activity can seemingly characterize the nonverbal decision-making. One can speculate that cognitive functions generally evolve across many time scales, from brief mental images to long-lasting trains of thought. Results of this work verified that transition from resting state to emotion-based or attention-based mental activity, and then the emergence of decision-making/preference cognition is accompanied by the presence of stochastic effects and high-dimensional character of the dynamics [35]. This dynamic was manifested by the self-similarity measure and variation in the long-range memory of the signal.

The proposed method additionally uses various extracted EEG measures to predict participants' choices. The prediction model based on statistical results of EEG analysis makes predictions of subjects' later choices and the products' out-of-sample and population success. Although the clustering algorithm in this work was so simple (since the focus of this research was mainly on characterization and not on classification or clustering), results were promising since the clusters were separated enough and also results were consistent with the self-report data. However, modification of the clustering algorithm presumably improves the prediction results for cases where prediction accuracy is requested.

Our result states that the perception and cognition that occurred in the brain by exposing to products' specifications can help redesign marketing strategies for a particular product that consumers might not choose due to cultural, social, and economic factors. For the selected product in this study, consumers may not be aesthetically attracted to this car due to its different appearance. This is the reason why we investigated the hypothesis of decision change owing to attentional stimuli. However, electric cars are becoming more and more like classic cars nowadays that may reduce the nonselection rate due to dislike. In this field, there are still some issues which are needed to be considered by psychological or neuromarketing researchers. Among these issues, we can mention changing driving behavior, mobility patterns, or charging routines that citizens encounter by adopting such cars. The present neuromarketing study could further be extended to unconscious control issues through BCI systems.

The results of experiments in this paper are in line with previous studies [40–43] and confirm that the neuromarketing platform can provide helpful information for marketers. Marketing has changed and morphed several times over the years, and neuromarketing has the potential to be the next breakthrough. Neuromarketing can effectively support traditional marketing claims and help marketers find better ways to introduce products to consumers effectively. However, unlike the straightforward nature of previous marketing techniques, neuromarketing is very challenging due to some reasons. The complexity of the decision-making process is controversial since many factors can affect human decision-making, of which some are not easily understandable. It is also needed to address ethical arguments for consumer free will. Finally, this method has not been easily accessible and acceptable for actual (not research) tests throughout society. However, researches addressing how to extend the experiment accomplishment in real-world situations on potential consumers and large-scale population are still demanded. Nowadays, technology improvement has provided wearable devices, including small and reliable EEG systems, for generating good signal quality and improving system performance; for example, new technologies suggest the usage of dry electrodes for reliable recording of brain activity [44]. Performing tests using such technologies can cause neuromarketing to be widespread and generate more realistic results. Another issue that researchers in neuromarketing and similar neuroscientific researches need to address is to validate their results within the selected population or even investigate and optimize the sample size for the test accomplishment. Since the results are derived from and based on the selected population, considerations on subjects and the number of subjects are needed. Based on the results of [45], a threshold of subjects for which the outcomes remained significant and comparable can be found. As a final note, randomizing the stimuli can also be considered. As we tried to address the usefulness of acknowledging the automobile attributes over all subjects, we did not randomize the stimuli in our experiment.

## 5. Conclusion

Studies in marketing neuroscience aim to open windows to consumers' brain activity and their behaviors in making decisions to buy a product. Knowing the actual market responses to advertise is the key to increasing sales and provides a complementary solution to the traditional marketing measures. This paper analyzed the impact of emotional and attentional stimuli on consumers' decision-making in a neuromarketing task. Neuroscience methods using EEG signals were applied to study a citizen's choice preference for an electric car. This study showed the effectiveness of the proposed neuromarketing framework in tracking cerebral activity. The results of neural marketing for a new product, which might not be adopted or even not be known by all community members, was successful and promising as it better fulfills consumers' demands. Future research can analyze valuation at various stages of the decision-making process and product price and design experiments to understand the circumstances that influence “choosing not to choose.” Furthermore, it would be interesting to examine the relationship between neurophysiological measures and market sales. Future works should also measure brain activity in relatively natural settings to achieve maximal generalizability to real-world situations.

## Figures and Tables

**Table 1:** Review and comparison of neuromarketing experimental tasks and their findings in the literature.

Reference	Modality/number of participants	Analysis method	Task	Aim	Activated region/dominant frequency bands
[22]	EEG/15	Power spectral density, statistical analysis, and logistic regression	Presenting images of shoes in 16 epochs of 10 s interval and taking like/dislike answer by button press	To determine discriminative EEG channels and frequencies in like/dislike decisions	In the LF band: frontal channel on the left (F7-A1) and a temporal channel on the right (T6-A2). In the HF band: central (Cz-A1) and occipital on the left (O1-A1) LF band (4 and 5 Hz)

[23]	EEG/20	Frequency analysis and LORETA analysis	Presenting five blocks of six different TV commercials, each lasting 30 seconds. Adds had different scores (better to worse)	To understand how brand perception influences when watching ads of the brands	Frontal cortex alpha, theta, and beta bands

[24]	EEG/15	Global field power	Presenting a long documentary lasted twenty minutes, interrupted by two advertising breaks	To assess the memorization of commercial clips	Prefrontal cortex and cingulate cortex theta

[10]	EEG/11	Power spectral density	Presenting a 30 min movie on neutral documentary interrupted by three commercial video clips	To test recalling the remembered video clips and assess emotional engagement by scoring pleasantness	Prefrontal and frontal cortex theta and alpha band

[25]	fMR/17	Statistical analysis	Presenting visual images of food and nonfood logs	To investigate the effect of branding and advertising (familiarity) on the preference of a products	Orbitofrontal, inferior prefrontal, and posterior cingulate cortex

[26]	MEG/16	Time series analysis	A shopping trip based on video footage of the interior of a supermarket for choosing consumer items	To study the temporal relationship of cerebral areas involved in consumers' choices and distinguish male/female subjects' choices during a simulated shopping	Left posterior cortices (women) and right temporal cortices (men); right parietal cortices for choosing the previously bought or used items and left inferior and right orbital cortices when selecting less-known items *γ*-oscillations

[27]	EEG/32	Deep learning classification	Presenting 40 music videos on scales of arousal, valence, and dominance, which were rated emotionally by participants	To bridge the gap between explicit and implicit consumer responses to marketing stimuli	Frontal alpha and beta

**Table 2:** Information and statistics related to the performed experiment.

Items in the questioner	Group A	Group B
Electric vehicle selection	16	29
Emotional selection	5	18
Attentional selection	11	11
Answer time length	(mean ± var) 2.76 + 1.32 s	(mean ± var) 3.87 + 1.61 s
Usefulness of the given information (yes/no)	13 yes	16 yes
Percentage of information usefulness (mean)	83	57
Change of decision to B when stimulated with specifications	0	5

**Table 3:** Significance value (*F* (*p*)) and effect size (*η*2) for ANCOVA test, investigating the age effect on groups' differentiation.

Variable	Group	Age	Group □age	Group □age *η*2
Frontal alpha mean power at stimulation stages	1.19 (0.06)	2.15 (0.1)	3.18 (0.04^*∗*^)	0.006
Central-parietal and central-frontal coherence	3.65 (0.7)	4.58 (0.3)	0.67 (0.9)	0.080
Frontal alpha DFA at decision-making stage	1.73 (0.02^*∗*^)	1.04 (0.08)	1.59 (0.7)	0.041

^*∗*^Significance at *p* < 0.05.

**Table 4:** Clustering results.

	Symbol	Accuracy (%)	PPV (%)	Test 1 *p* value	Test 2 *p* value
1	Frontal alpha band	81.2	84.7	0.3	0.4
2	Frontal-central alpha band	86.4	83.2	0.04	0.01
3	Frontal-central alpha and theta band	83.8	81.5	0.5	0.7
4	Frontal-central alpha band S1 and S2 stages	89.3	93.2	0.02	0.04
5	Frontal-central alpha band D1 and D2 stages	92.4	90.9	0.01	0.4

## Data Availability

Data are available upon request to the corresponding author.
